# Electronics with shape actuation for minimally invasive spinal cord stimulation

**DOI:** 10.1126/sciadv.abg7833

**Published:** 2021-06-25

**Authors:** Ben J. Woodington, Vincenzo F. Curto, Yi-Lin Yu, Héctor Martínez-Domínguez, Lawrence Coles, George G. Malliaras, Christopher M. Proctor, Damiano G. Barone

**Affiliations:** 1 Department of Engineering, University of Cambridge, Trumpington Street, Cambridge, UK.; 2 Department of Clinical Neurosciences, University of Cambridge, Cambridge Biomedical Campus, Cambridge, UK.; 3 Department of Neurological Surgery, Tri-Service General Hospital, National Defense Medical Center, Taipei, Taiwan.; 4 Tecnológico Nacional de México, Campus Morelia, Morelia, Mexico.

## Abstract

Spinal cord stimulation is one of the oldest and most established neuromodulation therapies. However, today, clinicians need to choose between bulky paddle-type devices, requiring invasive surgery under general anesthetic, and percutaneous lead–type devices, which can be implanted via simple needle puncture under local anesthetic but offer clinical drawbacks when compared with paddle devices. By applying photo- and soft lithography fabrication, we have developed a device that features thin, flexible electronics and integrated fluidic channels. This device can be rolled up into the shape of a standard percutaneous needle then implanted on the site of interest before being expanded in situ, unfurling into its paddle-type conformation. The device and implantation procedure have been validated in vitro and on human cadaver models. This device paves the way for shape-changing bioelectronic devices that offer a large footprint for sensing or stimulation but are implanted in patients percutaneously in a minimally invasive fashion.

## Teaser

Electronics that change shape inside the body enable keyhole surgery of spinal implants that can treat severe pain.

## INTRODUCTION

Spinal cord stimulation (SCS) is one of the most established neuromodulation therapies in clinical use. As of 2018, SCS devices were one of the most commonly used interventional neuromodulation techniques, with approximately 50,000 devices implanted annually (*1*). The first use of SCS was for the treatment of pain conditions, notably intractable back pain, but is now used to treat pain located in the legs, back, and chest, e.g., angina (*2*). It is estimated by the Centers for Disease Control and Prevention that, today, up to 8% of the U.S. population suffer from intractable back pain, that is, debilitating pain that is not responding to treatment by conventional methods such as nonsteroidal anti-inflammatory drugs or opioid drugs. SCS has now been used for several decades; however, despite its proven efficacy, cost-effectiveness, and advantages offered over conventional drug-based therapy, it remains neglected as a treatment by many general practitioners (*3*).

Patients undergoing SCS therapy will have electrodes implanted into the epidural space of the spinal column. The electrodes are usually connected to an implanted pulse generator, which controls the stimulation parameters. SCS devices are commonly separated into two categories: percutaneous linear-type probes and paddle-type probes, consisting of a one-dimensional (1D) and a 2D array of electrodes, respectively (*4*). With less than a 2-mm diameter, the cylindrical, linear design can be implanted percutaneously via a Tuohy needle in a relatively low-risk and simple “day surgery” procedure (*5*). Unfortunately, the benefit of simple implantation is negated by its decreased capability to manage pain because of its limited spatial range and likelihood of migration (displacement) when compared with a paddle-type probe (*6*). Implantation of up to three linear probes is possible to cover a larger area but generally remains less effective and more likely to migrate during its lifetime compared with a paddle-type device (*7*). Moreover, a linear electrode produces a spherical electric field of which only the part aimed toward the spinal cord is effective, leading to a reduced energy efficiency and limiting the capacity for a more targeted therapy (*8*).

Conversely, a paddle electrode, which is typically as wide as 12 mm, produces an electric field that is oriented toward the spinal cord only; thus, less power is required to obtain similar results (*3*). The array of forward-facing electrodes also allows for improved, more predictable targeting of electrical stimulation and a higher number of electrodes per device, typically between 16 and 32 (*9*). The flexibility in electrode configuration and the fixed relative position of the electrodes are likely the reasons these devices are commonly used in clinical investigations beyond the treatment of pain (*10*, *11*). The dominant drawback in using a paddle-type electrode is that a more invasive, higher-risk surgical procedure is required to implant the device (*9*). More recently, there has been interest within academic and clinical groups with regard to applying SCS to patients with spinal cord injury suffering from full or partial paralysis (*10*, *12*–*14*) or movement disorders within Parkinson’s disease (*15*).

While patients and carers are faced with a choice between paddle-type and percutaneous implants, recent technological developments in bioelectronics are changing the paradigm for the fabrication of implantable devices: This trend involves the use of techniques of the semiconductor industry to develop high-resolution microelectrode arrays made of thin films that are ultraconformable and include capabilities such as microfluidics for drug delivery. Examples include cortical arrays for recording brain activity at single neuron resolution (*16*) and spinal cord implants that can be used for rehabilitative therapy and are softer and more conformable than existing semirigid technologies (*17*, *18*). These devices are advancing our understanding of the neural networks (*19*, *20*) and are beginning to be translated to the clinic (*21*). At the same time, the field of soft robotics has yielded powerful paradigms of devices where shape is a dynamic property that can be changed using, e.g., pneumatic actuation (*22*–*24*). Devices with integrated fluidic channels are shown to be used as dynamic artificial organs and surgical manipulators. Here, we combine these two ideas to develop a minimally invasive paddle-type SCS (MI-SCS) comprising thin film electronics and integrated fluidics for mechanical actuation. The device can be rolled up to fit into a needle, allowing percutaneous implantation during a low-risk surgical procedure, and then expanded in situ to provide a paddle-type analog. This capability, transcends the duality of percutaneous versus paddle-type implants, offering the best of both worlds. It paves the way for implants in which shape is a dynamic property that can be manipulated by the clinician to minimize the invasiveness of neurosurgery and access high-performance form factors.

## RESULTS

### Design of the MI-SCS

**Fig. 1:**
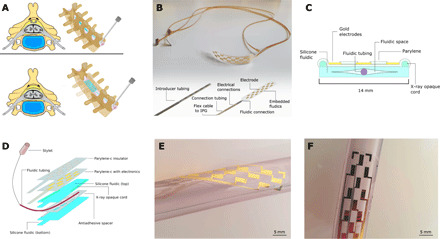
Design and fabrication of the MI-SCS. (**A**) A conceptual cross section of an implanted device within the spinal cord and a render of the device in its rolled and unrolled state within the spinal column. (**B**) A fabricated MI-SCS device before being packaged with the connections tube and secured inside the introducer. Inset is a device render with componentry annotated. (**C**) A cross-sectional diagram of the MI-SCS device. (**D**) An exploded illustrative view of the device architecture. (**E**) An optical image of the device wrapped helically around a tube to demonstrate inherent flexibility. (**F**) An optical image of the device positioned on a tube structure showing the representative electrode size. Photo credit: Ben Woodington, University of Cambridge.

MI-SCS was designed and fabricated using standard photolithography and soft lithography techniques. Components of the device intended to make contact with the patient were made entirely from biocompatible materials including parylene-C, silicone, polyethylene, polyimide, and gold. The MI-SCS functions as a paddle-type device analog once implanted, with the electrodes conforming to the surface of the dura but adapts to the dimensions of a percutaneous lead device during insertion. This is achieved by designing a fully rollable device from thin film electronics, which can be inserted via a percutaneous needle, and actuated with air pressure once implanted to transform into its functional, paddle-like orientation (Fig. 1A).

Once rolled, the device, and all packaging, including fluidic connections, electrical connections, and supporting tubing, was designed to be less than 2 mm in diameter to fit inside 14-gauge Tuohy needles, commonly used in percutaneous SCS lead placement. The packaging approach relied on concentric tubing, with the entire device enclosed in a polyimide tube, called the introducer. This protects the device as it is inserted into the body as well as assists in the navigation and reducing insertion trauma owing to its narrow footprint. A second smaller polyethylene tube contains the electronic connections and the fluidic tubing. The fluidic tube is the smallest and most nested of the tubes, allowing for connection between the embedded device fluidics and an external pump or syringe. The fluidic tubing acts additionally as a lumen for placement of a rigid stylet. During device insertion, a 400-μm stylet (Nuvectra, USA) is placed inside the fluidic tubing. This gives the device more mechanical stability and prevents kinking during insertion, an event that could damage the spinal cord or device. Electrical connections are made to the device using a custom thin, flexible cable, bonded using an anisotropic conductive film (ACF). The device design is shown in Fig. 1B.

Fabrication of the MI-SCS fluidic was achieved by creating a monolithic device using a process of incomplete silicone cure with a virtual fluidic created from an antiadhesive spacer. Under this method, the base layer of the fluidic does not fully cure before the second capping layer is added. Allowing the two layers to co-cure this way creates very strong cross-linked bonding, which results in a thin, robust device that is not prone to failure under pressure. Initially, fabrication of the device fluidics was explored using silicone-silicone bonding via O_2_ plasma activation, a common technique used in silicone fabrication (*25*, *26*), but this option does not enable the device to hold high-enough internal pressures. The bonding surface of the device was more prone to leak when under the amount of fluidic pressure required to unfurl the device. This motivated the move to create a monolithic silicone fluidic.

Using the techniques outlined, it was possible to fabricate the MI-SCS to a thickness between 30 and 60 μm, where device thickness is dominated by the silicone fluidic layer, with the electronic layer at only 4 μm (Fig. 1, C and D). During the MI-SCS device development, it was recognized that x-ray opacity was a key design requirement; this led to the placement of x-ray opaque markers, which were integrated during the soft lithography step as shown in Fig. 1D; these are staggered to allow the implanting clinician to infer the orientation of the device. Optical images of functional devices are shown in Fig. 1 (E and F). Figure 1E shows the intrinsic flexibility of the device owing to the thin films of silicone, parylene-C, and gold used for fabrication. Additional design and dimensional information is shown in fig. S1.

### In vitro validation

**Fig. 2:**
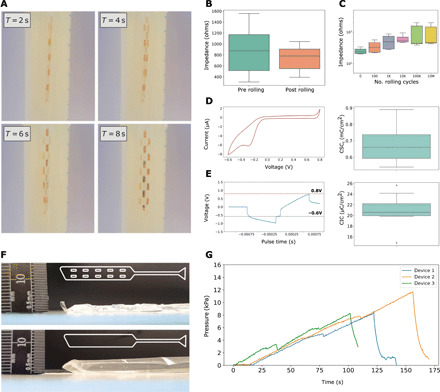
In vitro assessment of the device design. (**A**) Time series of an MI-SCS device being tested in an in vitro model to check for actuation and investigate whether air leaks are present. This model consisted of a latex balloon (Durex, UK) inflated with water inside of a secondary rigid container. Devices were actuated manually using a syringe. (**B**) Impedance recordings (1 kHz) from the MI-SCS (*n* = 9) before (preroll) and after (postroll) rolling and packaging the device inside of the introducer tubing. (**C**) Impedance reported at 1 kHz for MI-SCS device electrodes (*n* = 7) following a logarithmic increase in bending cycles. (**D**) A representative cyclovoltammetry (CV) recording from the device electrode A spread of the calculated cathodal charge storage capacity (CSC_c_) across three devices and eight electrodes is shown (*n* = 8). (**E**) A representative voltage transient charge injection curve is shown for the device electrode. A spread of calculated charge injection capacity (CIC) is shown for 11 electrodes across 3 devices (*n* = 11). (**F**) Image of a device showing maximum pneumatic expansion with a pillow-like fluidic structure (bottom) and a device with pillar-like structures engineered throughout the fluidic (top). The fluidic design is shown inset. (**G**) Time series graph of MI-SCS devices being inflated with air pressure at constant flow via a syringe pump until failure, as indicated by the sudden drop-off in pressure. Photo credit: Ben Woodington, University of Cambridge.

The devices were investigated in vitro for several features, including electrical stability, mechanical robustness, and dimensional changes following actuation of the fluidic component. Device unrolling was tested in a bench top model designed to simulate the epidural spinal cord space (Fig. 2A). The model consisted of a latex balloon inflated with water inside of a rigid container, simulating the virtual epidural space in vivo. The devices were inflated by hand using a syringe until they unfurled within the model. They were then placed in a bath of water, and pressure was added via a manual syringe while observing for any air bubbles leaking from the device.

The MI-SCS device was characterized for its electrical properties using electrical impedance spectroscopy techniques. First, rolling and packaging robustness was observed. The results show no increase in impedance during this rolling/unrolling cycle (Fig. 2B). Impedance was also used as an indicator of electrode functionality and yield. From the numerous impedance measurements carried out, an electrode yield of 80% was calculated.

Device stability for repeated bending was also determined; this was to ensure device integrity following a normal range of bending, which an implanted device would undergo. The number of sagittal plane bending cycles the human spine undergoes is varied but is estimated to be around 4400/day (*27*), the majority of these movements <10° in extent. The 10^7^ bending cycles carried out during this work would therefore represent approximately 6 years of mechanical stress in vivo. A gradual increase in impedance can be observed for the first 10^5^ cycles, after which the impedance plateaus. After 10^7^ bending cycles, the impedance remains within the expected range of 10^2^ to 10^3^ ohms (Fig. 2C). Charge injection capacity (CIC) limit and cathodal charge storage capacity (CSC_c_) characterization experiments were carried out for the device as shown in Fig. 2 (D and E). CIC measurements were carried out using a three-electrode system as reported in the literature (*28*, *29*). Voltage transients were observed and recorded using an oscilloscope. Increasing current was applied using a stimulation/recording device (Intan) until reaching gold water window limits; CIC was reported per unit area. CSC_c_ measurements were determined using a three-electrode setup and potentiometer. The cathodal portion of the cyclovoltammetry (CV) curves was integrated with respect to time to generate charge capacity; CSC was reported per unit area.

During early in vitro testing, an important clinical constraint was identified. This was the issue of what we have called anterior-posterior (AP) device expansion. This is where the device, when actuated, balloons out of plane rather than only expanding across its planar axis. If uncontrolled, the expansion in the AP dimension could put excessive pressure on the spinal cord, causing damage. A design restraint was established for the MI-SCS in which any fluidic actuation should not cause the device to excessively expand out of plane or balloon so that excess pressure is not placed on the spinal cord during device deployment. To limit this, pillar-like structures were engineered throughout the fluidic. Figure 2F shows the comparison between a pillow-like design initially conceived and a device used during the cadaveric validation of the MI-SCS. These expansion data are supported with a finite element model that explores device deformation and relative stresses on a device under pneumatic pressure using a reference material (figs. S2 and S3) (*30*).

After finalizing the fluidic design, the fluidic actuation of the device was assessed for pressure testing in triplicate (Fig. 2G). This is to ensure that the device does not fail during actuation in vivo. Devices were inflated at 1 ml/min using a syringe pump. Below the neck of the syringe, a Y-connector was used, which led to a pneumatic pressure sensor (shown in fig. S8). The devices were filled until failure, indicated by a sudden drop-off in pressure as shown in Fig. 2B. MI-SCS devices were able to hold between 8 to 12 kPa of pressure before failing.

### Human cadaveric validation

**Fig. 3:**
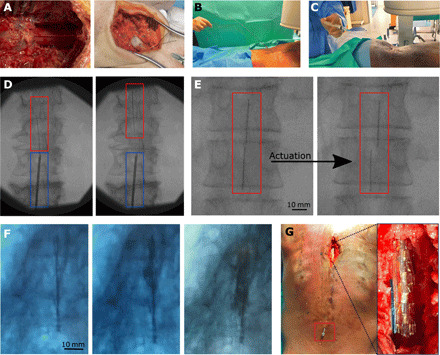
Human cadaveric assessment of the device and surgical procedure. (**A**) The spinal reconstitution procedure: The image on the left is how the dura is observed once exposed on the specimen following a laminectomy. The image on the right shows the surface of the dura following stable reconstitution with saline via the cervical spinal cord. (**B**) An MI-SCS device being placed on the spinal cord via a Tuohy needle with a stylet in place. (**C**) The stylet is removed, and the MI-SCS fluidic tubing is connected to a syringe, ready for actuation. (**D**) A fluoroscopy image of the device being guided rostrally along the spinal column. The blue box indicates the Tuohy needle location, and the red box highlights the MI-SCS device. (**E**) A series of fluoroscopy images: on the left, the device has been implanted and positioned but is in its rolled state, and on the right, the device has been actuated, as shown by a wider device profile; the darker vertical lines show the position of the fluoroscopy markers. (**F**) A time series of fluoroscopy images from an MI-SCS device inflated with an iodine-based contrast ink from within a human cadaver spinal column. From left (rolled) to right (unrolled). (**G**) A laminectomy and spinal cord exposure being performed once a device had been actuated; this was to directly observe the positioning of the implanted device. The main image shows the Tuohy needle at the lower section of the back. The image inset shows a device laying epidurally on the spinal cord. Photo credit: Mr. Christopher Constant, Evelyn Cambridge Surgical Training Centre.

After successful in vitro validation, human cadaveric models were used to test the proposed surgical approach of fully packaged MI-SCS devices. A spinal cord model was established by reconstituting the subdural space with saline. A fluidic tube was secured within the spinal cord, and a flow pump was used to perfuse the spinal cord with constant pressure. This was to reduce the epidural space and simulate a clinically relevant environment. Figure 3A shows how, immediately following exposure, the spinal cord is dehydrated, and a large epidural space remains (left). Following stable perfusion, the spinal cord appears brighter and wider (right), and the epidural space is reduced to an anatomically representative size.

The devices were implanted at the L3/4 vertebra (Fig. 3B) by a trained neurosurgeon operating as close to a percutaneous device implantation procedure as was possible, i.e., through a Tuohy needle, without performing a laminectomy at the device insertion site. Each device was then connected to a syringe (Fig. 3C) ready to be actuated using air by the operator.

MI-SCS devices enclosed within a polyimide introducer tube were inserted through a 14-gauge Tuohy needle and navigated up the spinal column (S3), centrally, on the posterior side of the spinal cord (Fig. 3D). Once in location, as indicated by fluoroscopy, they were deployed onto the dura (Fig. 3E). Device unrolling was validated by fluoroscopy imaging initially, although some devices were validated using a liquid, iodine-based radio-opaque contrast agent (Fig. 3F). Although this did not offer a perfectly clear image via fluoroscopy, an optimized method could be considered in the future. Last, a full spinal cord laminectomy was performed at the site of implantation (Fig. 3G).

## DISCUSSION

In this work, we have leveraged developments in thin film bioelectronics and soft robotics to demonstrate a flexible, shape-changing, paddle-type spinal cord stimulator that can be inserted percutaneously. Our approach involves active, on-demand shape actuation and represents a paradigm change to previously reported passive shape-changing materials or injectable devices. We show that it is possible to introduce such a device to a human cadaver using standard percutaneous procedures and expand the device into its paddle conformation in a clinically relevant location on the spinal cord. When deployed, the device expands to the dimension of common, clinically available paddle SCS devices, with a width as large as 14 mm. This is comparable to the popular Medtronic 5-6-5 device, which has a width of 12 mm, while potentially offering far fewer surgical risks and a day surgery procedure, which would involve a simple epidural needle insertion.

The limitations of x-ray imaging proved to be a challenge in the placement of thin film bioelectronic devices guided by fluoroscopy. Several techniques and materials were explored to improve the opacity of the device under fluoroscopy. Our strategy to solve this is the incorporation of bismuth microparticles into a silicone matrix, which we mold into markers. These devices can be seen under fluoroscopy following standard U.K. radiography practices. The inherent softness of silicone allows for the device to be packaged within the introducer tube, a small sub-2-mm space. Patterning with this material could be explored in the future to further aid device insertion and positioning.

Device robustness, yield, and functionality were of crucial interest for this work. Device failures did present themselves; however, this was overwhelmingly due to fluidic failure as opposed to electrical failure. As the devices required relatively high pressure to be manipulated in vivo, any pinholes or tears in the silicone chamber would result in nonfunctional devices. We aim to optimize this process to improve on device yield and consistency in the future.

The validation of this device concept was carried out on human cadaver models. The intention behind this was to validate the underlying mode of operation for the device and to test its mechanical capability. Furthermore, spinal cord stimulators with electrodes of similar dimension have been long accepted as offering clinical benefits to patients suffering from pain conditions. Hence, in vivo animal testing was not justified at this stage of development. Owing to the relative size of the device, being designed for human treatment, and difficulties in scaling the device to very small sizes suitable for murine models, large-mammal models would need to be used to carry out further validation. In future work, we aim to carry out these experiments to further demonstrate the safety profile of this device, its utility in treating pain, and the long-term chronic acceptance of such a device.

Here, we have shown that by combining photo- and soft lithography fabrication methods, we can create a clinically relevant paddle-type SCS device with the advantages of being thin and flexible enough to be implanted through minimally invasive means. Although we have applied the device to a spinal cord stimulator matching commonly used dimensions of existing paddle-type devices, we envisage a device that could cover a much larger area while retaining a small insertion footprint, offering a new paradigm for central nervous system interfaces. Devices leveraging this kind of shape-changing fluidic actuation in situ could be applied to other stimulating or recording devices where low-risk surgery is preferred, such as on the brain or retina or in hard-to-reach parts of the body, for example, deep peripheral nerves or organ interfaces. Using this shape-adaptive technology, we will be able to place large-area electrical interfaces throughout the body using only low-risk, keyhole-like, and minimally invasive surgical approaches. In addition to shape change, the combination of bioelectronics and soft robotics can yield implants with unique properties such as self-motility inside the body, revolutionizing the way we practice neurosurgery.

## MATERIALS AND METHODS

### Study design

The hypothesis behind this study was that by integrating fluidic channels into thin, flexible electronic devices, we could create a new class of neuromodulation device. This device could be implanted through a small surgical site, i.e., a needle channel, and actively expanded by a clinician into a large-area electronic device at the site of interest. With SCS as the target indication, we sought to combine the advantages of both percutaneous lead– and paddle-type devices currently clinically available.

Our first goal was to design these devices and test them within an in vitro environment, developing the best form of fluidic actuation and optimizing fabrication methodologies to produce a robust device, thin enough to package inside a standard Tuohy needle.

Our second goal was to test the robustness of these electronics undergoing the type of mechanical stresses one would expect during the packaging and clinical use of the device throughout its lifetime. A secondary aim was to test the failure threshold of the device when undergoing inflation.

The third goal was to validate the device in a clinically relevant model, notably using human cadavers. Following model development, we sought to demonstrate that the device could be implanted using standard percutaneous surgical approaches, imaged using standard equipment (e.g., fluoroscopy) and actively deployed using pneumatic pressure in situ.

Mechanical/electrical characterization was performed for *n* = 9 and *n* = 7 electrodes for packaging and chronic bending testing, respectively. Charge storage and charge injection experiments were performed on *n* = 8 and *n* = 11 electrodes, respectively, sampled across three devices at random. None of the experimental work or data analysis was performed blinded.

### Fabrication of MI-SCS

A visual representation for the fabrication of the MI-SCS is provided in fig. S4. The fluidic portion of the device was created by soft lithography forming of silicone (Nusil MED-6015, Polymer Systems UK). The base-layer silicone was first diluted with hexane at a 50% ratio. The organic solvent was used to reduce the viscosity of the uncured silicone (*31*, *32*) before spin coating and resulted in a thinner final device. Spin coating was carried out at 1500 rpm for 90 s to form 10-μm-thick layers. A 6-μm-thick parylene antiadhesive spacer was placed following a brief (5 to 10 min) partial curing step at 60°C to form the fluidic channel. A capping layer of silicone was added by blade coating using a 23-μm-thick spacer. During the formation of the silicone fluidic, x-ray markers in the form of bismuth powder (mesh 100, Sigma-Aldrich, UK)–infused silicone cords were integrated along the flanks of the device. The device was then treated using a silane solution (A174, Thermo Fisher Scientific, UK) and coated with a 1-μm-thick parylene-C layer.

The electronics were fabricated from gold using standard photolithographic processing using a plastic photomask (JD Photo Data). AZ5214E photoresist (Merck) was spin coated onto the wafers at 4000 rpm for 30 s. This was followed by a baking step on a hot plate at 110°C for 120 s. After the substrates returned to ambient temperature, they were exposed to ultraviolet (UV) (7 s, 80 mJ/cm^2^) using a mask aligner (Karl Suss Contact Mask Aligner MA/BA6), and this was followed by a second baking step at 110°C for 120 s. The substrates were allowed to cool for several minutes before being flood exposed with UV light (20 s, 80 mJ/cm^2^). The substrates were allowed to cool and developed in AZ741 (Merck)/deionized (DI) water (1:4) for 38 to 40 s; this was then rinsed with DI water and air dried with compressed nitrogen. Each sample was inspected with a Zeiss Axio Scope (A1) to ensure complete development.

The substrate was activated with O_2_ plasma under vacuum (60 s at 100 W and 0.6 mbar O_2_) using a PlasmaPro 80 reactive ion etching (RIE). Using a Lesker e-Beam evaporator (PVD 75), titanium (Ti) and gold (Au) were deposited to produce the electronic components of the device. First, 10-nm Ti was evaporated under vacuum (<6 × 10^−6^ torr). The substrate was allowed to rest under vacuum for 10 min before 100-nm Au was deposited. Liftoff was performed with acetone, the substrates were allowed to soak for 30 min, and they were then sprayed with acetone from a bottle to encourage the remaining material to be removed. Last, the substrates were washed with isopropyl alcohol (IPA) and inspected under an optical microscope to ensure complete liftoff. If any traces were shorted due to incomplete liftoff, these were further treated with acetone.

Following a second parylene deposition, photolithography was repeated to expose electrodes and contact pads using AZ9260 (Merck), spin coated for 3000 rpm for 30 s, and baked at 120°C for 120 s. They were then UV-exposed for 20 s and developed in AZ726MiF (Merck) for 6.5 min. The substrate was then etched using RIE [Oxford Plasma Pro 80 RIE, 160 W/O_2_ 50 standard cubic centimeter per minute (sccm)/CHF_3_ 5 sccm] until metal exposure was complete.

The devices were packaged using custom-built polyimide/copper flexible cables (Printed Electronics, UK) and a Fintech bonder using ACF (Jetro, Japan). The polyethylene fluidic connector tubing [Porex 0.4-mm inside diameter (ID)/0.8-mm outer diameter (OD), Scientific Laboratory Supplies Ltd, UK] was placed by hand and fixed by injecting a small volume of 2-part epoxy resin (RS Pro, UK) around the connector. The tip of the tubing was sealed with epoxy to ensure the stylet did not pierce the device fluidic during insertion. The tube was perforated with small air holes to allow airflow into the fluidic during actuation.

All electrical and fluidic connections were packaged inside a connections tubing (Pellathane 55D, Nordson Medical, USA), which was sealed with a small amount of epoxy (RS Pro, UK). The entire device was then rolled and placed inside the outer introducer tubing (Reinforced PI, Nordson Medical, USA). A small amount of silicone oil (Nusil MED-450, Polymer Systems, UK) was used to lubricate the final introducer packaging step to reduce stress and damage on the device. This process is illustrated in fig. S5.

### Electrical characterization

To investigate the electrical characteristics of the device, three-point impedance measurements were taken before and after expected device stresses. Impedance measurements can shed light on whether the electrodes or interconnects are being damaged during these processes. All impedance measurements were taken using an AutoLAB potentiostat, a phosphate-buffered saline (PBS) solution (0.01 M, Sigma-Aldrich), and a platinum electrode was used at the electrode interface.

Impedance measurements were taken on the device before rolling into the sheath at nine electrode locations. These devices were then rolled into their sheath tubing and actuated to encourage unrolling on the bench; the second impedance spectroscopy measurements were taken at the sampling sites. Impedance was measured between 10^5^ and 10^0^ Hz and reported at 1 kHz.

CSC of the device was determined using three-point CV techniques. Measurements were carried out using an AutoLAB potentiostat with a PBS solution (0.01 M, Sigma-Aldrich) used as electrolyte. A platinum electrode was used as the counter electrode, while an Ag/AgCl electrode was used as a reference. CV measurements were carried out using a 0.1-V sweep rate and 0.00244-V step within a window of −0.6 to 0.8 V, the established water window for gold. A minimum of six cycles were performed for each measurement to allow the recording to settle, and only the final recorded cycle was analyzed.

The CIC of the device was determined using a stimulation source and measured voltage transients using an oscilloscope. An Intan RHS 128-channel stim/recording controller and an RHS2000 32-channel headstage were used to connect to the device and provide a charge-balanced current stimulation pulse with a phase width of 633.3 μs and an interphase delay of 100 μs. Platinum probes were used as both counter and reference electrodes, and a PBS solution (0.01 M, Sigma-Aldrich) was used as electrolyte. The pulse was gradually increased in amplitude while observing the voltage transient trace from a Keysight DSOX1204G oscilloscope. When the cathodic interphase transient reached −0.6 V, the current amplitude was recorded.

As the MI-SCS would be situated on the spinal cord in clinical practice, it was necessary to assess the mechanical robustness of the device, mainly concerned with the electrodes and/or interconnects fracturing. A custom rig was designed and built to hold devices on a flexible bed; either end of this bed was attached to a stress/strain cycler capable of applying push or pull forces on a device. The push-pull motion caused a bending in the bed, which bent the device. The bed was programmed to implement a full-bend cycle every 2 s to varying angles between 5° and 45°. Impedance spectroscopy measurements were taken from seven electrodes of the device, representing top, middle, and bottom portions of the device, after 0, 10^2^, 10^3^, 10^4^, 10^5^, 10^6^, and 10^7^ bending cycles. Impedance was measured between 10^5^ and 10^0^ Hz and reported at 1 kHz using a platinum reference and counter electrode, and a PBS solution (0.01 M, Sigma-Aldrich) was used as electrolyte.

### Device pressure characterization

MI-SCS devices were connected to a 60-ml syringe pump (KD Scientific) via a 3D-printed t-connector, which led to a pneumatic pressure sensor (NXP, MXP5100GP), interpreted by an Arduino Mega (fig. S8). The syringe pump was programmed to dispense air at 1 ml/min. Devices were inflated while the system pressure was measured. When a sudden pressure drop was recorded, indicating device failure, the testing was stopped.

### Establishing cadaveric model

The MI-SCS devices were tested during three separate of human cadaver sessions at the Evelyn Surgical Training Center, Cambridge (UK). Three fresh-frozen specimens were used—two male and one female. The male specimens were used during the first two sessions and the fourth session, whereas the female specimen was used during the third session. The model was first established. To achieve the spinal reconstitution with saline, the dura was first exposed by incision above the cervical part of the spinal cord, followed by the removal of spinous process and full laminectomy at the C4 to C6 vertebrae. An incision was made in the dura with a 18-gauge needle; a polyethlye tubing (OD = 1.4 mm) was inserted into the incision and secured using a purse string suture (Prolene 4-0), and a small amount of cyanoacrylate glue (Locktite) was dispensed around the tube to ensure an adequate seal. The tubing was connected to a flow pump (Flowsteady, model 200). The pump was set to 140 mmHg at a rate of 1.5 liters/min. After 30 s, the dura could be seen to expand, and after 60 s, liquid could be seen emerging from the epidural space, indicating either a leak of the dura or fluid displacement as the dura expanded. The pump was reduced to a pressure of 70 mmHg and 0.5 liter/min. The seal was monitored for around 30 min before the first implantation to ensure that the seal held, and the dura did not leak or expand further.

### Device implantation

The devices were implanted through a 14-gauge spinal needle (Hamilton, PN:7749-03, Ga14/100 mm), and a reinforced polyethylene introducer (Pellathane 65D, Nordson Medical) was used. A stylet was used (Nuvectra, USA) to rigidify the device during insertion. During device insertion, images were taken laterally by a fluoroscope (fig. S7). The devices were guided rostrally along the spinal column on the dural surface. Once the device had been navigated to an appropriate position, the stylet was removed, the outer introducer tubing was retracted, and air pressure provided via a manual Luer-lock syringe was used to actuate and unfurl the device. During the insertion of the device and during actuation, both still and live images were recorded using a fluoroscope (Siemens Siremobil compact L) operated by a professional radiographer. Images were captured between 80 and 88 kV.

### Investigation of suitable x-ray strategy and fabrication of x-ray markers

Several x-ray opacity markers were explored, with the use of silicone cords infused with bismuth powder being the most effective. These various preparations were first screened using a veterinary x-ray machine (fig. S6). The materials were placed either freestanding within the machine or below a canine vertebra for contrast. The x-ray was operated by a trained veterinary radiographer and used within the power boundaries of normal operation. Although not analogous to fluoroscopy and through the trunk of a human, the veterinary machine allowed the screening of various materials and the observation of their relative opacities using a similar technology. All bismuth-infused silicones were prepared in-house. These were cast using custom molds, which were 3D printed (Asigna UV cured, Detax). A 2:1 mixing ratio of bismuth powder (mesh 100, Sigma-Aldrich, UK) to polydimethylsiloxane (Dow Corning 184, 10:1) was used. The optimal thickness was 450 μm, although markers between 300 and 1000 μm were investigated.

## SUPPLEMENTARY MATERIALS

Supplementary material for this article is available at http://advances.sciencemag.org/cgi/content/full/7/26/eabg7833/DC1

## Appendix

View/request a protocol for this paper from *Bio-protocol*.
